# IL-23 and IL-17A are not involved in hepatic/ischemia reperfusion injury in mouse and man

**Published:** 2015-12-17

**Authors:** Pim B. Olthof, Rowan F. van Golen, Megan J. Reiniers, Milan Kos, Lindy K. Alles, Martinus A. Maas, Joanne Verheij, Thomas M. van Gulik, Michal Heger

**Affiliations:** 1 Department of Experimental Surgery, Academic Medical Center, University of Amsterdam, Amsterdam, the Netherlands; 2 Department of Pathology, Academic Medical Center, University of Amsterdam, Amsterdam, the Netherlands

**Keywords:** liver damage, sterile inflammation, innate immunity, cytokines, oxidative stress, mouse model, clinical trial

## Abstract

**Background:**

Hepatic ischemia and reperfusion (I/R) is common in liver surgery and transplantation and compromises postoperative liver function. Hepatic I/R injury is characterized by sterile inflammation that contributes to hepatocellular necrosis. Many immune cells and cytokines have been implicated in hepatic I/R injury. However, the role and relevance of IL-23 and IL-17A remains controversial in literature. **Aim**: To determine whether the IL-23/IL-17A signaling axis is activated in hepatic I/R using a triple-level experimental approach (in vitro, in vivo, and clinical).

**Methods:**

IL-23 and IL-17A were assayed by ELISA in the supernatant fractions of cultured murine (RAW 264.7) macrophages that were activated by supernatant fractions of necrotic cultured mouse (AML12) hepatocytes. Similarly, levels of these cytokines were determined in plasma samples and liver tissue of mice (N = 85) subjected to partial (70%) liver I/R. Finally, IL-23 and IL-17A were assayed in plasma samples obtained from a controlled cohort of liver resection patients who were either subjected to I/R (N = 27) or not (N = 13).

**Results:**

Activated macrophages did not produce IL-23 in response to supernatant of necrotic AML12 hepatocytes. IL-23 and IL-17A were not elevated in mice subjected hepatic I/R and were not elevated in serum from patients subjected to I/R during liver resection.

**Conclusion:**

IL-23 and IL-17A are not involved in hepatic I/R injury in mouse and man.

**Relevance for patients:**

If IL-23 and IL-17A were to mediate hepatocellular injury following I/R, these cytokines would constitute potential therapeutic targets. Since this study has revealed that IL-23 and IL-17A do not play a role in hepatic I/R, other pathways and therapeutic targets should be considered when developing modalities aimed at reducing hepatic I/R injury.

## 1. Introduction

Hepatic ischemia/reperfusion (I/R) injury is encountered in various medical situations, including liver resection, liver transplantation, and circulatory shock. Irrespective of the cause, I/R injury produces a sterile immune response during which innate and adaptive immune cells induce liver damage. With the advent of immunotherapy, the hepatic I/R immune response has been extensively studied in order to identify molecular substrates that can be targeted pharmacologically to attenuate I/R-induced liver injury. One of the signaling cascades that has received notable attention is the IL-1β/IL-23/IL-17A axis, which plays a role in I/R injury of the kidney [1], brain [2], and heart [3]. The role of the IL-1β/IL-23/IL-17A signaling axis in hepatic I/R injury has been investigated previously, but the available data are currently equivocal and elusive. For example, IL-17A knockout (KO) mice exhibited less hepatocellular necrosis and lower transaminase levels after 90 min of partial hepatic ischemia compared to wild-type controls [4]. Neutrophil invasion was greatly reduced in IL-17A knockout mice, suggesting that IL-17A is a key regulator of neutrophil-mediated tissue damage following hepatic I/R [4]. The role of IL-17A in I/R was also demonstrated in a mouse model using 60-min partial hepatic ischemia. However, that study found that natural killer (NK) cells instead of neutrophils were the source of IL-17A [5]. Moreover, the increase in hepatic IL-23 levels was suggested to promote IL-17A production. A later study identified unconventional RORγ-expressing T cells as the source of IL-17A in a murine 90-min partial liver ischemia model [6]. In addition, Tan et al. [7] reported that RORγ^+^ neutrophils drive hepatic I/R injury via an IL-1β/IL-23/IL-17A signaling axis, based on findings in a murine partial hepatic I/R model and plasma samples from 7 patients subjected to hepatic I/R. In contrast, minor-to-no increases in IL-17A and IL-23 were measured during reperfusion in wild-type mice subjected to 60 min of 70% hepatic ischemia [8]. Only mice lacking the transcription factor interferon regulatory factor 3 exhibited an increase in IL-17A and IL-23 [8]. This last study therefore ruled out an involvement of IL-17A and IL-23 in hepatic I/R injury.

The diverging results generated using different models of hepatic I/R currently preclude the drawing of uniform conclusions and adequate clinical translation [9]. Essentially, several scenarios could activate IL-23/IL-17A signaling during surgery-induced sterile inflammation. The cytokines IL-1β and IL-23 can activate RORγ-positive innate immune cells either directly or in concert with damage-associated molecular patterns (DAMPs) derived from necrotic hepatocytes. DAMPs are innocuous intracellular molecules that gain immunogenic properties when released from dying cells, and have recently been identified as most proximal triggers of I/R injury [10,11]. Innate immune cells subsequently produce IL-17A within hours after stimulation, thereby driving neutrophil accumulation [12]. Taking these considerations into account, the aim of this study was to determine the involvement of IL-1β/IL-23/IL-17A signaling in hepatic I/R. Using in vitro DAMP transfer experiments, a validated murine hepatic I/R model, and plasma samples from a controlled patient cohort exposed to liver I/R, it is shown that the IL-1β/IL-23/IL-17A axis is not causally linked to liver I/R injury.

## 2. Materials & Methods

### 2.1. Damage-associated molecular pattern transfer assay

The mouse hepatocyte cell line AML12 was cultured in William’s E (WE) medium (Lonza, Basel, Switzerland) supplemented with 10% (v/v) fetal bovine serum (FBS, Thermo Fisher Scientific, Waltham, MA), penicillin (100 U/mL, Thermo Fisher Scientific), streptomycin (100 μg/mL, Thermo Fisher Scientific), insulin (5 µg/mL, Sigma-Aldrich, St. Louis, MO), L-glutamine (2 mM, Thermo Fisher Scientific), and hydrocortisone hemisuccinate (50 µM, Sigma-Aldrich). Due to the large quantity of hepatocytes required for the DAMP transfer experiments, AML12 cells were used instead of primary mouse hepatocytes. RAW 264.7 murine macrophages were cultured in Dulbecco's Modified Eagle Medium (DMEM) (Lonza) supplemented with 10% FBS, penicillin (100 U/mL), and streptomycin (100 μg/mL). Both cell lines were maintained at standard culture conditions (37 °C, humidified atmosphere composed of 95% air and 5% carbon dioxide). AML12 cells were transferred to hydrocortisone-free medium 1 day prior to the experiments to prevent glucocorticoid-induced suppression of macrophage function following medium transfer. For all assays, RAW 264.7 cells were subcultured in 24-wells plates at a density of 5 × 10^5^ cells per well and used in experiments when 80% confluence was reached.

To stimulate macrophages with hepatocyte-derived DAMPs, full T162 culture flasks containing roughly 18 × 10^6^ AML12 cells were rendered necrotic in 3.5 mL of WE medium by heat shock. The heat shock entailed 1-h incubation at 60 ºC (7 × 7-cm hotplate, VWR International, Radnor, PA) followed by 24-h incubation at standard culture conditions. The extent of heat shock-induced AML12 cell death (> 95%) was confirmed in pilot experiments using trypan blue exclusion assays on the cell suspension. After 24 h, the culture medium containing necrotic AML12 cells, and hence DAMPs, was either used directly or centrifuged for 5 min at 400 × g to remove cellular debris. DAMP-rich medium (500 µL/well) was subsequently transferred to RAW 264.7 cells at ratios of approximately 50:1 or 25:1 necrotic AML12 cells per macrophage (e.g., the amount of necrotic AML12-enriched medium roughly contains 50 or 25 AML12 cells per RAW 264.7 macrophage). Heat-shocked WE medium was used as negative control. Lipopolysaccharide (100 ng/mL final concentration) in heat-shocked WE medium was used as positive control.

### 2.2. Macrophage activation: inducible nitric oxide synthase activation

To determine the extent of macrophage activation following incubation with DAMP-rich medium, nitrite was measured in the culture supernatant of RAW 264.7 cells using the Griess Reagent System Kit (Promega, Cat # G2930, Madison, WI) according to the manufacturer’s protocol. Macrophage activation coincides with the activation of inducible nitric oxide synthase (iNOS) [13], leading to the production of nitric oxide (•NO), which is rapidly converted to the stable metabolite nitrite that can be used as an indirect measure of •NO. Absorbance was measured at 540 nm on a Synergy HT luminescence plate reader (BioTek Instruments, Winooski, VT). Values were normalized to protein content per well (Pierce BCA Total Protein Assay Kit, Thermo Fischer Scientific).

### 2.3. Macrophage activation: production of reactive oxygen species

To detect extracellular production of reactive oxygen species (ROS) by RAW 264.7 macrophages, the fluorogenic, cellimpermeable probe 2’7’-dichlorodihydrofluorescein (DCFH_2_) was prepared from the parent compound 2’7’-dichlorodihydrofluorescein diacetate (DCFH_2_-DA, Molecular Probes/Life Technologies, Eugene, OR) as described previously [14]. DCFH_2_ was dissolved in DMSO at a 50-mM concentration. The non-fluorescent DCFH_2_ is oxidized by ROS [15] to the highly fluorescent dichlorofluorescein (DCF). The extent of DCF fluorescence in this setup is hence a measure of the degree of extracellular oxidant formation.

RAW 264.7 cells were seeded and incubated with DAMP-rich medium, prepared as described in section 2.1, at a 50:1 hepatocyte:macrophage ratio. After 24-h incubation, cells were washed once with PBS, after which 500 µL of 50 µM DCFH_2_ in PBS (37 ºC) was added per well. Phorbol 12-myristate 13-acetate (PMA, 0.5 μL of 2 mM PMA in DMSO, 2 μM final concentration, or solvent control) was included as positive control. DCF fluorescence (*λ*_ex_ = 460 ± 40 nm and *λ*_em_ = 520 ± 20 nm) was measured in time-based acquisition mode (t = 20 min at 2-min intervals) in a temperature-controlled microplate reader (37 °C; Synergy HT, BioTek Instruments). Data were corrected for fluorescence in cell-free wells (reflecting DCFH_2_→DCF auto-oxidation) and total protein content. Data were expressed as fold increase relative to baseline fluorescence (i.e., directly after addition of PBS).

### 2.4. Macrophage activation: NF-κB assay

The RAW 264.7 NF-κB/LUCPorter (Novus Biologicals, Littleton, CO) cell line is a stably transfected RAW 264.7 murine macrophage cell line that expresses an optimized Renilla luciferase reporter gene (RenSP) under the transcriptional control of an NF-κB response element.

RAW 264.7 NF-κB/LUCPorter macrophages were seeded in 24-well plates at a density of 2.0 × 10^5^ cells/well and cultured under standard conditions to 70% confluence during 24 h. Cells were washed with PBS and incubated with 0.5 mL of either 100 ng/mL LPS in WE medium (positive control) or DAMP-rich medium (described in section 2.1) for 6, 12, or 24 h at standard culture conditions. Next, the cells were lysed using Cell Culture Lysis Reagent (Promega, Madison, WI) and the cell lysates were transferred to 96-well plates (Greiner Bio-One, Kremsmünster, Austria) for the luciferase reporter assay or protein determination. The luciferase activity of the cell lysates was measured with a luciferase reporter system (Promega) according to the manufacturer’s instructions. Bioluminescence was measured in a microplate reader (Synergy HT, BioTek Instruments) and data were corrected for protein content (Pierce BCA Total Protein Assay Kit, Thermo Fisher Scientific).

The NF-κB assay was repeated with DAMPs derived from cells that had been subjected to 24-h ischemic incubation. Growth medium of AML12 cells was replaced with 3.5 mL of fresh WE medium. The cells were purged with nitrogen, the cap was sealed, and cells were incubated for 24 hours at standard culture conditions. The supernatant of AML-12 cells was collected and either centrifuged or used as is without centrifugation. Supernatants were stored at 4 °C until use for RAW 264.7 macrophage stimulation.

### 2.5. Macrophage IL-23 production

In the DAMP stimulation assay, IL-23 was measured after 24 h in culture supernatant by ELISA according to the manufacturer’s instructions (Quantikine ELISA kit, R&D Systems, Minneapolis, MN). Data were corrected for protein content (Pierce BCA Total Protein Assay Kit, Thermo Fisher Scientific).

### 2.6. Mouse model of hepatic ischemia/reperfusion

Male specific pathogen-free C57BL/6J mice (N = 85) weighing between 25-30 g were obtained from Charles River (JAX stock number 000664, L’Arbresle, France). The animals were housed in a temperature-controlled cabinet (21 ºC) with a 12-h dark-light cycle and ad libitum access to CRM pellet food (Special Diets Services, Essex, UK) and water. All animals were acclimated for 7 days before inclusion in the study. All experimental protocols were approved by the Institutional Review Board.

Hepatic ischemia was induced for 30, 60, or 90 min and followed by 1-24 h of reperfusion and compared to animals subjected to sham procedure. All surgical and experimental details of the in vivo I/R experiments have been described in detail previously [9,16]. At the indicated time points, the animals were anesthetized and sacrificed by exsanguination using cardiac puncture.

### 2.7. Sample collection and processing

Blood was collected in heparin-anticoagulated microtainers (BD Biosciences, Franklin Lakes, NJ) and centrifuged for 10 min at 10,000 × g. ALT was measured by the Department of Clinical Chemistry using a Cobas 8000 modular analyzer (Roche, Basel, Switzerland). After exsanguination, part of the left liver lobe was fixed in 10% (vol/vol) formalin solution (J.T. Baker, Center Valley, PA) for 2 to 4 days, after which the samples were dehydrated and embedded in paraffin, cut to 5 µm-thick sections, and stained with hematoxylin and eosin (H&E). Confluent necrosis was scored in a blinded fashion by an experienced hepatopathologist (J.V.).

For transcriptomic analysis, 30 mg of left liver lobe tissue was stored in RNAlater (Qiagen, Venlo, the Netherlands) overnight at 4 ºC and subsequently at –20 ºC until RNA isolation.

### 2.8. RNA extraction, PCR arrays, and qRT-PCR

Total RNA was isolated from ± 30 mg of mouse liver tissue with the RNeasy minikit (Qiagen) as described previously [17], albeit with minor modifications. One µg of RNA was reversetranscribed to cDNA using the Transcriptor First Strand cDNA Synthesis Kit (Roche Applied Sciences, Penzberg, Germany) according to the manufacturer’s instructions. PCR arrays (Th17 for Autoimmunity and Inflammation PCR Array, RT2 Profiler PCR Array, Qiagen) were performed as described in [17].

Data were processed according to Ruijter et al. [18]. PCR efficiencies for each amplicon were calculated and amplicons with an efficiency of < 1.80 or > 2.00 were excluded from further analysis. All samples were normalized to the housekeeping genes that showed the most stable expression over all arrays (i.e., hypoxanthine-guanine phosphoribosyltransferase, HPRT). Data were expressed as fold increase relative to the sham group.

For RT-qPCR of in vitro samples, TNFα, IL-1β, and IL-6 primers were designed with NCBI Primer Blast to span an exon-exon junction, ordered from Life Technologies, and dissolved to a concentration of 5 µM in nuclease-free water. The primers nucleotide sequences were as follows: TNFα, forward 5’-ACAGAAAGCATGATCCGCGA-3’, reverse 5’-TCTGA-GTGTGAGGGTCTGGG-3’; IL-1β, forward 5’-TGCCACC-TTTTGACAGTGATG-3’, reverse 5’-GCCACCTTTTGACA-GTGATGAG-3’; IL-6, forward 5’-GCCTTCTTGGGACTG-ATGCT-3’, reverse 5’-TGCCATTGCACAACTCTTTTCT-3’; and HPRT, forward 5’-CCTGGCGTCGTGATTAGTGA-3’, reverse 5’-GGGCTACAATGTGATGGCCT-3’. Quantitative reverse transcriptase polymerase chain reaction was performed on a LightCycler 480 (Roche, Basel, Switzerland) using a reaction volume of 10 µL, consisting of 2 µL of cDNA (i.e., 25 ng), 2 µL of nuclease-free water (Qiagen), 1 µL of primer mix (0.5 µM final primer concentration), and 5 µL of SensiFAST SYBR No-ROX mix (Bioline, London, UK). Melting curve analysis and ethidium bromide-stained agarose gel electrophoresis were used to validate primer specificity. Data were processed as described [18], normalized to the housekeeping gene HPRT, and are expressed as fold increase relative to the sham group.

### 2.9. Quantification of cytokines in murine plasma and liver tissue

Murine plasma IL-23 and IL-17A concentrations were determined in heparin-anticoagulated plasma samples by ELISA according to the manufacturer’s instructions (Quantikine ELISA kits, R&D Systems). Liver samples were homogenized in meta-phosphoric buffer (pH = 6.0) and centrifuged at 4000 × g for 10 min. IL-17A concentrations were determined in the supernatant by ELISA and corrected for protein content (Pierce BCA Total Protein Assay Kit, Thermo Fisher Scientific).

### 2.10. Study subjects

Between January 2012 and December 2014, blood samples were collected from 40 patients scheduled for a major liver resection (> 3 Couinaud segments [19]) as part of an observational study registered with http://clinicaltrials.gov under identifier NCT01700660. The study design was described previously [20]. Patient characteristics are shown in Table 1. The experimental protocol was approved by the institutional review board (protocol # 2012_238) and written informed consent was obtained from all participants.

### 2.11. Quantification of human plasma cytokines

Human IL-23 and IL-17A concentrations were determined by ELISA (Duoset ELISA kits, R&D Systems) in heparin-anticoagulated samples and expressed per mg of plasma protein (Pierce BCA Total Protein Assay Kit, Thermo Fisher Scientific) to correct for peroperative hemodilution [22].

### 2.12. Statistical analysis

Intergroup differences were tested with a Mann Whitney U or Kruskall-Wallis test. For all in vitro and mouse data, the experimental groups were compared to the medium/sham group. For clinical data, a Friedman test was used to examine intragroup differences over time and a Mann Whitney U test was employed to assess intergroup differences at individual time points. A p-value < 0.05 was considered statistically significant. Statistical analysis was performed using GraphPad Prism (GraphPad Software, La Jolla, CA).

## 3. Results

### 3.1. Hepatocyte-derived damage-associated molecular patterns activate macrophages

In light of the most plausible route of IL-23/IL-17A activation outlined in the introduction, the first aim was to evaluate whether murine macrophages produce IL-23 upon stimulation with hepatocyte-derived DAMPs, which is an experimental setting analogous to the in vivo situation.

**Figure 1.**
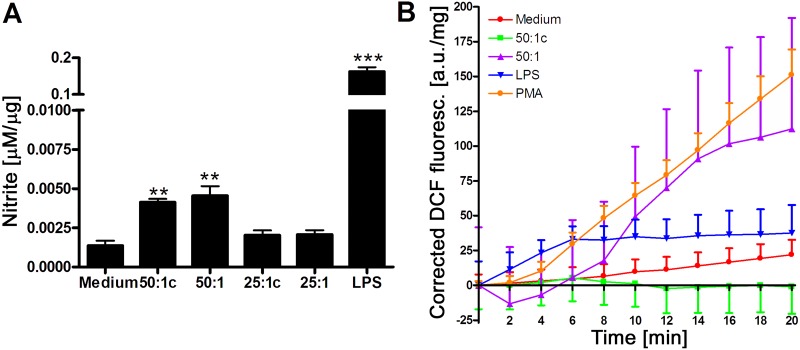
A: Nitrite production after 24-h incubation of RAW 264.7 cells with AML12 hepatocyte-derived DAMPs. Data are plotted as mean ± SEM with N = 7-8 per group. B: Real-time ROS production after 24-h incubation of RAW 264.7 cells with AML12-derived DAMPs. Data are plotted as mean ± SEM with N = 4 per group. * indicates *p* < 0.05, ** indicates *p* < 0.01, and *** indicates *p* < 0.001 compared to the medium group. Abbreviations: c, centrifuged; LPS, lipopolysaccharide; PMA, phorbol 12-myristate 13-acetate; DCF, dichlorofluorescein.

Stimulation of murine RAW 264.7 macrophages with AML12 hepatocyte-derived DAMPs resulted in macrophage activation, as demonstrated by the formation of the •NO degradation product nitrite (Figure 1A) and the extracellular formation of ROS (Figure 1B). Macrophage activation was observed when macrophages were stimulated at a 50:1 cell ratio (AML12: RAW 264.7), but was absent at a 25:1 cell ratio, indicating that substantial parenchymal injury is required to induce innate immune activation. Removing cellular debris from DAMP-rich culture medium by centrifugation prior to medium transfer reduced macrophage activation (Figure 1), suggesting that both large-sized cell remnants (pelleted by centrifugation) and molecular DAMPs (not pelleted by centrifugation) are involved in innate immune activation following hepatocyte necrosis.

### 3.2. Hepatocyte-derived damage-associated molecular patterns induce the production of TNFα, IL-1β, and IL-6 but not IL-23 in an NF-κB-independent manner

**Figure 2.**
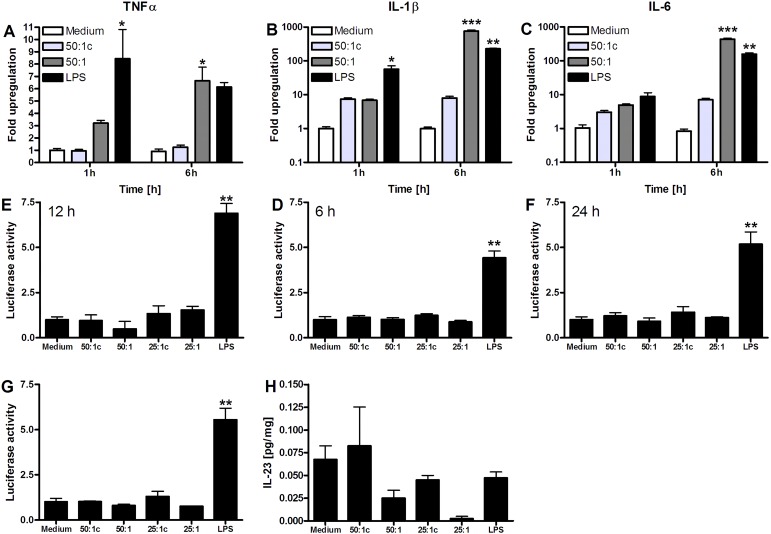
Pro-inflammatory signaling by DAMP-exposed RAW 264.7 macrophages. A: TNFα, B: IL-1β, and C: IL-6 mRNA expression after 1-h and 6-h DAMP incubation. All results are presented as fold upregulation compared to medium incubation (N = 3-4 per group). D-G: Luciferase reporter assay of RAW 264.7 NF-κB/LUCPorter cells following medium-, DAMP-, or LPS stimulation after D: 6 h, E: 12 h, and F: 24 h (N = 3 per group). G: Luciferase reporter assay after stimulation with DAMPs derived from ischemia-subjected necrotic cells, medium, or LPS after 24 h of exposure (N = 3 per group). Luciferase activity is expressed as the fold change relative to control. H: IL-23 production by murine macrophages in response to AML12 hepatocyte-derived DAMPs measured by ELISA in RAW 264.7 cell supernatant and corrected for protein (N = 4 per group). All data represent mean ± SEM. * indicates *p* < 0.05, ** indicates *p* < 0.01, *** indicates *p* < 0.001 compared to the medium samples.

Stimulation of RAW 264.7 cells with DAMPs resulted in expression of the proinflammatory cytokines TNFα, IL-1β, and IL-6 (Figure 2A-C) independent of the transcription factor NF-κB. (Figure 2D-F) The NF-kB experiments were repeated using DAMPs from AML12 cells derived by ischemic necrosis with similar results (Figure 2G). Despite the DAMP-induced macrophage activation (Figure 1), RAW 264.7 cells did not produce IL-23 in response to DAMP stimulation (Figure 2H).

**Table 1:** Patient characteristics. Plasma samples were collected from 40 consecutive patients (Pt.) that were scheduled for a resection of 3 or more Couinaud liver segments [19]. As the decision to use vascular inflow occlusion and thus induce ischemia/reperfusion (I/R) injury was made during surgery on the basis of the operative course, patients were non-randomly assigned to either the I/R group (N = 27) or the control group (N = 13). The cumulative ischemia time was reached by continuous (-c) or intermittent (-i) occlusion of the afferent hepatic blood vessels [21].

Pt.	Gender	Age	Diagnosis	I/R	Cumulative ischemia time [min]	Resected segments [Couinaud]
1	M	46	Perihilar cholangiocarcinoma	Yes	50-i	I − IV
2	F	57	Perihilar cholangiocarcinoma	No	-	II − IV
3	F	49	Hepatocellular adenoma	Yes	50-i	V − VIII
4	M	57	Perihilar cholangiocarcinoma	Yes	50-i	I + V – VIII + part IV
5	F	65	Proximal cholangiocarcinoma	Yes	43-i	I − IV
6	M	69	Intrahepatic cholangiocarcinoma	Yes	56-i	I − IV
7	F	67	Perihilar cholangiocarcinoma	Yes	20-c	I − IV
8	M	26	Metastasized melanoma	Yes	65-i	I − IV
9	F	33	Focal nodular hyperplasia	No	-	II − IV
10	M	65	Colorectal liver metastases	Yes	40-c	VII − VIII*
11	M	62	Chronic sclerosing cholangitis	No	-	II − IV
12	M	53	Hepatocellular carcinoma	No	-	II − IV
13	M	70	Perihilar cholangiocarcinoma	Yes	42-i	V − VIII
14	M	58	Hepatocellular carcinoma	No	-	V − VIII
15	M	63	Colorectal liver metastases	Yes	120-i	II − IV + wedge IV + VII
16	F	75	Intrahepatic cholangiocarcinoma	No	-	I − IV
17	M	71	Perihilar cholangiocarcinoma	No	-	I + V − VIII + part IV
18	F	46	Hepatocellular adenoma	Yes	52-c	II + V + part VIII
19	M	56	Colorectal liver metastases	Yes	76-i	V − VIII
20	M	76	Perihilar cholangiocarcinoma	Yes	39-i	I − IV
21	F	71	Chronic/recurring cholangitis	Yes	24-c	V − VIII
22	M	68	Intrahepatic cholangiocarcinoma	Yes	26-i	II − IV
23	F	72	Perihilar cholangiocarcinoma	Yes	68-i	I − IV
24	M	18	Hepatoblastoma	No	-	II − IV
25	M	68	Hepatocellular carcinoma	Yes	93-i	I − IV + part VIII
26	M	46	Perihilar cholangiocarcinoma	Yes	68-i	V − VIII
27	F	63	Intrahepatic cholangiocarcinoma	No	-	I − IV
28	M	36	Chronic/recurring cholangitis	No	-	II − IV
29	M	70	Colorectal liver metastases	Yes	46-i	V − VIII + part IV
30	M	67	Perihilar cholangiocarcinoma	Yes	20-c	V − VIII
31	M	66	Hepatocellular carcinoma	Yes	72-i	VII − VIII + I
32	F	54	Hepatocellular carcinoma	Yes	43-i	II − IV
33	F	76	Intrahepatic cholangiocarcinoma	Yes	33-c	II − IV
34	F	70	Perihilar cholangiocarcinoma	No	-	I − IV
35	M	78	Perihilar cholangiocarcinoma	Yes	30-c	V − VIII + I
36	M	65	Colorectal liver metastases	No	-	V − VIII
37	M	75	Epithelioid hemangioendothelioma	No	-	V − VIII
38	M	66	Perihilar cholangiocarcinoma	Yes	68-i	I − IV
39	M	43	Perihilar cholangiocarcinoma	Yes	25-c	V – VIII + I
40	M	65	Colorectal liver metastases	Yes	68-i	I – IV + wedges V/VI/VIII

* Patient 10 had a segment VII-VIII resection of colorectal liver metastases. Although less than 3 segments were resected, the patient was included in the analysis because the 40 min of continuous ischemia used during surgery likely induced substantial I/R injury.

### 3.3. IL-23 and IL-17A do not play a role in ischemia/reperfusion-induced liver injury in mice

**Figure 3.**
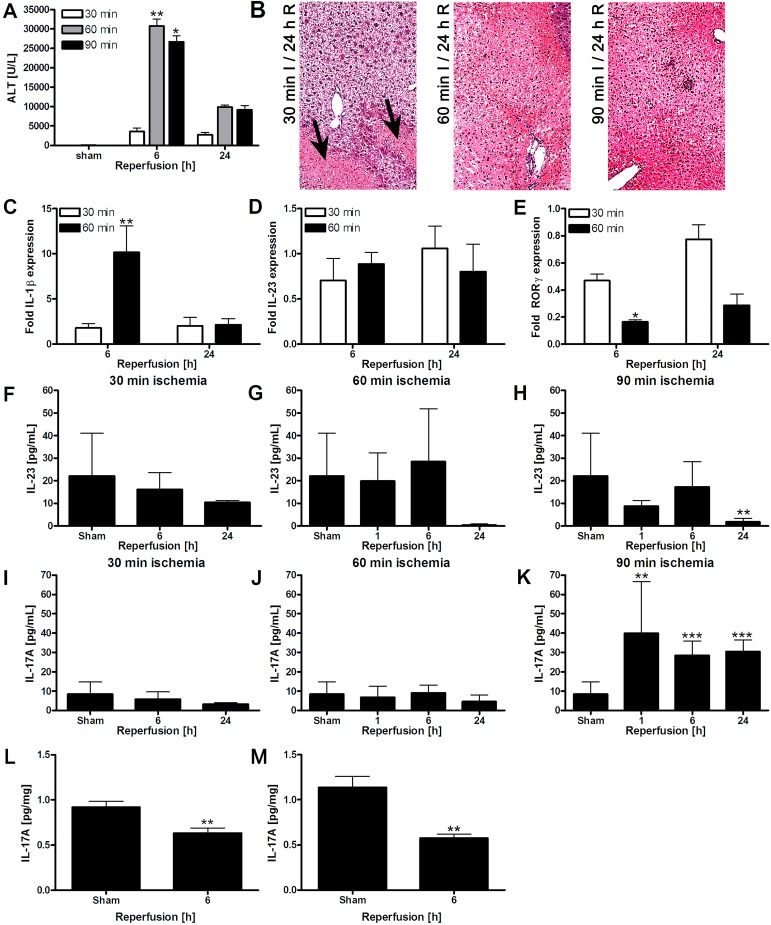
A: ALT levels during reperfusion following different ischemia times (N = 6-8 per group). B: H&E staining of livers subjected to 30-90 min of ischemia and 24 h of reperfusion. After 24 h, 20% necrosis was observed after 30 min ischemia (black arrows) versus 75-100% after 60-90 min ischemia. C-E: Liver IL-1β, IL-23, and RORγ mRNA expression following 30 or 60 min of ischemia and 6 or 24 h of reperfusion. Data are expressed as fold upregulation compared to the sham group (N = 3-5 per group). F-H: Plasma IL-23 levels determined by ELISA after 30, 60, or 90-min ischemia (N = 12 for sham and N = 4-9 for I/R groups). I-K: Plasma IL-17A protein levels after 30, 60, or 90 min of ischemia (N = 12 for sham and N = 4-9 for I/R groups). L: IL-17A protein levels after 60 min of ischemia in liver tissue homogenates (N = 8-10 per group). M: IL-17A protein levels after 90 min of ischemia in liver tissue homogenates (N = 6-9 per group). All data represent mean ± SEM.* *p* < 0.05, ** *p* < 0.01, and *** *p* < 0.001 compared to the sham group.

The second goal was to study activation of the IL-1β/IL-23/IL-17A axis in a partial (70%) hepatic ischemia model in mice, using either 30 or ≥ 60 minutes of ischemia to induce moderate or severe hepatocellular injury, respectively (Figure 3A-B). In these models, plasma IL-1β was elevated only at 6 h of reperfusion after 60-min ischemia (Figure 3C). However, no increases in liver IL-23 mRNA (Figure 1D), RORγ mRNA transcripts (Figure 1E), or IL-17A mRNA were observed (undetectable, data not shown).

Moreover, there was no elevation of circulating IL-23 protein levels after 24 h of reperfusion in any of the injury models (Figure 3F-H), and no alterations in hepatic IL-23 protein levels were observed at 1 h and 6 h of reperfusion (Figure 3L). Corroboratively, plasma IL-17A protein levels did not increase after 30 or 60 min of ischemia (Figure 3I-J). Although an elevation in systemic IL-17A protein was observed after 90 min of ischemia (Figure 3K), this trend could not be confirmed in liver tissue homogenates (Figure 3L and M).

### 3.4. IL-23 and IL-17A do not contribute to post-ischemia/ reperfusion liver damage in surgical patients

In order to validate the in vitro and in vivo results, blood samples were collected from forty consecutive patients scheduled for a major liver resection (i.e., ≥ 3 Couinaud liver segments). Patients were non-randomly assigned to either the control or I/R group based on the intra-operative decision on whether or not to use vascular inflow occlusion (i.e., induce I/R).

**Figure 4.**
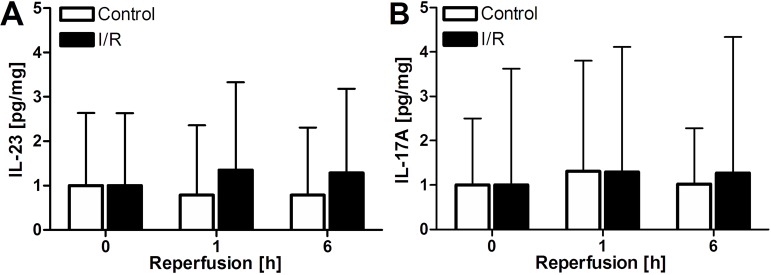
A-B: Plasma IL-23 and IL-17A levels, corrected for plasma protein, in patients subjected to liver resection with or without I/R (N = 13 for control and N = 27 for I/R group). * *p* < 0.05, ** *p* < 0.01, and *** *p* < 0.001 compared to the sham group. All data represent mean ± SEM.

In this patient cohort, plasma IL-23 and IL-17A levels were measured at baseline (i.e., prior to surgery) and 1 h and 6 h after I/R (I/R group) or removal of the resection specimen (control group). In line with the animal results, there was no increase noted in IL-23 (Figure 4A) or IL-17A (Figure 4B) following major liver resection in patients, irrespective of the use of I/R.

## 4. Discussion

We examined activation of the IL-1β/IL-23/IL-17A axis following liver I/R and demonstrated that there is no clinically relevant role for IL-23 and IL-17A in the pathogenesis of hepatic I/R injury. This conclusion is supported by in vitro experiments, data generated with a validated mouse model, and results from a controlled clinical cohort.

Although hepatic IL-23 expression following I/R has been reported previously [23], the cellular source of this cytokine has remained elusive. In sterile inflammatory disorders like I/R injury, IL-23 is likely produced by liver-resident antigen-presenting cells in response to pro-inflammatory stimuli such as DAMPs [24]. To emulate this scenario, murine macrophages were activated by mouse hepatocyte-derived DAMPs, which resulted in the production of ROS and cytokines, but not IL-23. DAMP-induced macrophage activation occurred independent of the transcription factor NF-κB, which supports the earlier notions that innate immune activation after liver I/R pro ce eds v ia a TL R4-IRF3 ax is ra ther th an th e MyD88-dependent TLR-4-NF-κB route [8,25,26], thereby preventing DAMP-mediated IL-23 transcription [27].

Because it cannot be ruled out that IL-23 is produced by a different leukocyte subset (e.g., dendritic cells) or that IL-23 is generated in a DAMP-independent manner, the IL-1β/IL-23/IL-17A signaling axis was further explored in moderate (i.e., 30-min ischemia) and severe (i.e., ≥ 60-min ischemia) mouse hepatic I/R injury models, defined on the basis of ALT and hepatocellular necrosis profiles [9]. In these models, no role for IL-23 and IL-17A was found, which corroborates the in vitro data. Furthermore, one of the proximal triggers of the axis, IL-1β, was only upregulated in the severe but not the moderate injury model, suggesting that extreme pathological conditions would be required for IL-23 and IL-17A production, which remained absent. As the severe injury model is difficult to translate to the clinical situation due to the extensive necrosis (> 75%) observed in these animals [9], results obtained in the moderate injury model might better reflect the clinical situation. The absence of IL-1β in the moderate injury model therefore adds weight to the irrelevance of the IL-1β/IL-23/IL-17A-axis in hepatic I/R injury.

The most important step in research is clinical translation of in vitro and in vivo data. No changes in plasma IL-23 or IL-17A protein levels were observed 1 or 6 h after surgery in both study arms. It is thus debatable whether the increase in IL-17A measured previously in a very small clinical cohort is attributable to hepatic I/R. Considering that there was no control group in the study of Tan et al. [7], the rise in plasma cytokines could have resulted from other factors not properly accounted for in the experimental design. Thus, in order to accurately determine the effect of hepatic I/R on plasma cytokine levels, measurements should be derived from a controlled cohort subjected to liver surgery with or without I/R, as reported here. The large data variability (Figure 4), which is inherent to the nature of our study, further underscores the need for including an appropriate control group.

## 5. Conclusions

There is no apparent or clinically relevant role of IL-23 and IL-17A in hepatic I/R injury. These findings are based on negative results generated in vitro, in vivo, and clinical studies. Accordingly, IL-23 and IL-17A are not instrumental in post-ischemic liver inflammation and injury. Other pathways and therapeutic targets should be considered when developing modalities aimed at reducing hepatic I/R injury.
